# Bristol Medical Care

**Published:** 1984-10

**Authors:** Meg Baskett, Sarah Lee

**Affiliations:** Executive Officers, Bristol Medical Care Ltd.; Executive Officers, Bristol Medical Care Ltd.


### Bristol Medical Care

**Meg Baskett, S.R.N. and Sarah Lee**
Executive Officers, Bristol Medical Care Ltd.

Successful companies are born of ideas which are able to fill a gap hitherto unprovided for. What started off as an idle discussion abour patients from abroad seeking treatment in the United Kingdom gradually gelled into the proposal to set up a company to arrange high quality medical treatment and at the same time provide a caring service to look after all those potential problems and worries that must upset a patient arriving in this country. We envisaged someone who had perhaps never before been to the United Kingdom with language difficulties, unsure of local travel facilities, hotels etc. and generally frightened about their health. We felt there was a need for a service to meet the patient as he or she arrived in England, establish a friendly trust and guide him through the complicated maze of this country's facilities and customs as well as assisting him to the best medical care at value-for-money cost.

Bristol as the principal medical centre in the South West with specialist experts in all branches of medicine seemed an ideal centre to base such a project. Communications were excellent and a first class private ambulance service, based in Bristol with a branch at Heathrow, was available for really ill patients. Others could be met using an ordinary car of varying opulence according to the patients wishes or could come by train. The overall costs would undoubtedly be less than the equivalent facilities in the capital city.

The plunge was taken, brochures and note paper were printed, a telex number was obtained and arrangements were made with hotels, travel agents, interpreters etc. We called our company Bristol Medical Care.

We started by writing to over 100 Bristol based consultants to let them know of our service and to seek their willingness to see and treat our patients. Everyone who replied was most enthusiastic in their response which was very encouraging. One consultant raised the question of the ethical position of the project which had two consultants as directors of the company. However, we were reassured by the Chairman of the B.M.A. Ethical Committee who, after seeking legal advice, was able to inform us that no transgression of professional ethics would occur if the advertising of the service was confined to the medical profession.

To establish Bristol Medical Care we clearly had to contact doctors abroad who refer patients to Britain. The research for this proved to be an enormous task but after hours in the reference library we compiled the following list.

1. Medical Directors of 115 hospitals and clinics in the Gulf States.
2. Medical Officers of 250 British based companies in the Middle East.
3. Medical Attachés at 25 Embassies in London.
4. Ministers of Health in all of the target countries.
5. Medical Directors of all the major airlines.
6. Medical Directors of international rescue services.

We wrote to these individuals informing them of our existence and our aims to provide the highest standards of medical care at realistic prices, coupled with our complete service for all the patients needs from their arrival in Britain until they left the country.

Bristol has two excellent private hospitals in the Chesterfield and St. Mary's, both of which have superb operating theatres, which just manage to cope with present demand for private treatment. In 1986 the new United Medical Enterprises Hospital will open, which will offer high technology in the form of CT scanning and complete radiology, intensive care and the ability to undertake cardiac and neuro-surgery in addition to the normal facilities. This is likely to attract, amongst others, many more foreign patients to Bristol. Bristol Medical Care will be well established by that time and will hopefully be able to help to attract these patients.

In summary, the services provided by us include—

1. Arranging International flights.
2. Meeting patients at the airport on arrival.
3. Transport by ambulance or car as appropriate.
4. Supplying interpreters as required.
5. Arranging hotel accommodation of appropriate grade to suit the patient.
6. Arranging appointments for medical consultants.
7. Arranging hospitalisation as required.
8. Arranging cultural and special diets.
9. Arranging social and shopping trips for relatives.
10. Providing local environmental information.
11. By prior arrangement a package including flights, transport within the U.K., accommodation and medical treatment etc can be obtained.

### CASE HISTORY

The following anonymous case history may illustrate how Bristol Medical Care has helped two of its patients.

Mr. and Mrs. Smith came to Britain from Australia to visit their son who was seconded to the British Navy. Mrs. Smith had developed a malignant melanoma with cerebral, pulmonary and bony secondaries. She was aware of her poor prognosis but very much wanted to see her son again. She had also heard of the Bristol Cancer Help Centre, and was prepared to seek treatment there as a last hope.

After two weeks as an inpatient at the Bristol Cancer Help Centre Mrs. Smith was admitted to the Bristol Homeopathic Hospital with sudden partial loss of vision, severe headaches and continual vomiting. Bristol Medical Care was contacted to arrange for her to be flown home to Sydney with her husband. She clearly required a stretcher throughout the long journey and a nurse to administer the dexamethazone and diamorphine that she had now been prescribed to be given in conjunction with the homeopathic medication and special diet. The patient on a stretcher, her husband, and the nurse required a total of nine seats on the plane, which were not easy to obtain at short notice during peak travel periods. The British Airways Senior Flight Medical Officer was contacted and fortunately turned out to be a doctor who had studied anaesthesia in Bristol in 1970. As such she was naturally most helpful and arranged medical clearance for the patient and the necessary drugs within 24 hours. She did however explain that should the patient die during the flight her body would have to be off loaded at Bombay! This horrifying potential complication was carefully considered but on balance it was decided that the risk of such an event occurring at this stage was slight.

Wings Paramedic Ambulance Service was engaged to transport the patient to Heathrow with a State Registered Nurse of sister status who was to care for the patient throughout the 36 hour journey.

Two days after contacting Bristol Medical Care Mr and Mrs. Smith were booked to leave for Australia. Mr. Smith was taken to collect his tickets from the travel agent. Having done so he then informed us that he feared he would be unable to undertake the long journey because of an embarrasing medical problem! He had been unable to make a private appointment to see a consultant in Plymouth in less than three weeks to look into this ailment. After gentle questioning it transpired that he had developed acute retention of urine and was in enormous discomfort. In 10 minutes we arranged an appointment with a consultant urologist during his lunch break. After sedation with diazepam and relief of pain by temgesic, a urinary catheter was passed which relieved the problem which was due to prostatitis.

The couple left Bristol 4 hours later in the ambulance for Heathrow, to catch the 2200 hours British Airways flight to Sydney. Mrs. Smith's condition slowly improved during the flight because of the substantial doses of steroids and adequate pain control. She was able to walk off the plane at Sydney. Her husband's condition also improved and fortunately the nurse did not need to insert the urinary catheter she had in her pocket.

